# Pulmonary vasculitis in Hughes-Stovin syndrome (HSS): a reference atlas and computed tomography pulmonary angiography guide—a report by the HSS International Study Group

**DOI:** 10.1007/s10067-021-05912-3

**Published:** 2021-09-17

**Authors:** Yasser Emad, Yasser Ragab, Cal Robinson, Sonia Pankl, Pablo Young, Marianna Fabi, Parag Bawaskar, Ossama Ibrahim, Doruk Erkan, Bhupen Barman, Jasna Tekavec-Trkanjec, Balakrishnan Jayakrishnan, Michael Kindermann, Melek Kechida, Aurelien Guffroy, Rafael S. Silva, Faten Frikha, Alaa Abou-Zeid, Maged Hassan, Harrison W. Farber, Mohamed H. Abdelbary, Leticia Tornes, Jason Margolesky, Nashwa El-Shaarawy, Sami Bennji, Manoj Kumar Agarwala, Ahmed Saad, Taoufik Amezyane, Sergio Ghirardo, Vitor Cruz, Bruno Niemeyer, Khalfan Al-Zeedy, Hamdan Al-Jahdali, Natalia Jaramillo, Serkan Demirkan, Issam Kably, Jung Tae Kim, Johannes J. Rasker

**Affiliations:** 1 Rheumatology Department, Faculty of Medicine, Cairo University, Kasr Al-Ainy St., Cairo, 11562 Egypt; 2 Radiology Department, Faculty of Medicine, Cairo University, Kasr Al-Ainy St., Cairo, 11562 Egypt; 3 Department of Paediatrics, Division of Nephrology, The Hospital for Sick Children, Toronto, ON Canada; 4 Servicio de Clínica Médica, Hospital Británico de Buenos Aires, Perdriel 74, C1280 AEB, Buenos Aires, Argentina; 5 Pediatric Cardiology and Adult Congenital Unit, S. Orsola-Malpighi Hospital, University of Bologna, 40138 Bologna, Italy; 6 Department of Cardiology, Topiwala National Medical College & B.Y.L Nair Charitable Hospital, Dr. A.L. Nair Road, Mumbai, 400008 Maharashtra India; 7 Morecambe Bay University Hospitals Lancaster, Ashton Rd., LancashireLancaster, LA1 4RP UK; 8 Barbara Volcker Center for Women and Rheumatic Diseases, Hospital for Special Surgery, Weill Cornell Medicine, New York, NY 10021 USA; 9 Department of General Medicine, North Eastern Indira Gandhi Regional Institute of Health and Medical Sciences (NEIGRIHMS), Mawdiangdiang, Shillong, 793018 Meghalaya India; 10 Department of Pulmonary Medicine, Dubrava University Hospital, AvenijaGojkaŠuška 6, 10000 Zagreb, Croatia; 11 Department of Medicine, Sultan Qaboos University Hospital, 123, Al-Khoud, Muscat, Oman; 12 Innere Medizin III (Kardiologie/Angiologie), Universitätskliniken Des Saarlandes, Kirrberger Straße, 66421 Homburg/Saar, Germany; 13 Internal Medicine and Endocrinology Department, Fattouma Bourguiba University Hospital, University of Monastir, Rue du 1er juin 1955, 5019 Monastir, Tunisia; 14 Service D’immunologieclinique et Médecine Interne, Centre de Référence des Maladies Auto-Immunes Systémiquesrares (RESO), hôpitauxuniversitaires de Strasbourg, nouvelhôpital civil, 67091 Strasbourg, France; 15 UFR Médecine Strasbourg, Université de Strasbourg, 67000 Strasbourg, France; 16 Unidad de Enfermedades Respiratorias, Hospital Regional de Talca, Calle 1 Norte 1990, Talca, Chile; 17 Department of Internal Medicine, HediChaker Hospital, 3029 Sfax, Tunisia; 18 Public Health Department, Faculty of Medicine, Cairo University, Kasr Al-Ainy St., Cairo, 11562 Egypt; 19 Chest Diseases Department, Faculty of Medicine, Alexandria University - Al Kartoom Square, Al Azareta, Alexandria, 21526 Egypt; 20 Division of Pulmonary, Critical Care and Sleep Medicine, Tufts University School of Medicine, Boston, MA USA; 21 Department of Radiology, Badr Hospital, Faculty of Medicine, Helwan University, Helwan, Egypt; 22 Department of Neurology, University of Miami Miller School of Medicine, Professional Arts Center, 1150 NW 14th St., Suite 609, Miami, FL 33136 USA; 23 Rheumatology and Rehabilitation Department, Faculty of Medicine, Suez Canal University, Ismailia 4.5 Km the Ring Road, Ismailia, 41522 Egypt; 24 Division of Pulmonology, Department of Medicine, Tygerberg Academic Hospital/Stellenbosch University, Francie van Zijl Drive Tygerberg 7505, Cape Town, South Africa; 25 Department of Cardiology, Apollo Hospitals, Jubilee Hills, Hyderabad, 500096 India; 26 Internal Medicine Department, Faculty of Medicine, Cairo University, Kasr Al-Ainy St., Cairo, 11562 Egypt; 27 Department of Internal Medicine, School of Medicine, Mohammed V Military Teaching Hospital, Mohammed V-Souissi University, Rabat, Morocco; 28 Clinical Department of Medical, Surgical and Health Science, University of Trieste, Piazzale Europa, 1, 34127 Trieste, TS Italy; 29 Serviço de Reumatologia, Hospital das Clínicas, Faculdade de Medicina, Universidade Federal de Goiás, Goiânia, GO Brazil; 30 Departamento de Radiologia, Instituto Estadual do Cérebro Paulo Niemeyer, R. do Rezende, 156 - Centro, Rio de Janeiro, RJ 20231-092 Brazil; 31 Pulmonary Division, Department of Medicine, King Saud University for Health Sciences, King Abdulaziz Medical City, Riyadh, 11426 Saudi Arabia; 32 Cardiology Department, Hospital Puerta de HierroMajadahonda, C/Joaquin Rodrigo 3, 28222 Madrid, Spain; 33 Department of Dermatology and Venerology, Faculty of Medicine, Izmir KatipÇelebi University, Karabağlar, Izmir Turkey; 34 Department of Radiology, Section of Vascular and Interventional Radiology, Jackson Memorial Hospital, University of Miami Miller School of Medicine, Miami, FL USA; 35 Department of Cardiovascular and Thoracic Surgery, Cheonan Chungmu Hospital, 8 Dagamal 3-gil Seobuk-gu, Cheonan-si, Chungcheongnam-do Republic of Korea; 36 Faculty of Behavioral, Management and Social Sciences, Department Psychology, Health and Technology, University of Twente, Drienerlolaan 5, 7522NB Enschede, The Netherlands

**Keywords:** Classification of HSS pulmonary vasculitis, Computed tomography pulmonary angiography (CTPA), HSS reference atlas, Hughes-Stovin syndrome (HSS), Pulmonary artery aneurysm

## Abstract

**Introduction:**

Hughes-Stovin syndrome (HSS) is a systemic vasculitis characterized by widespread venous/arterial thrombosis and pulmonary artery aneurysms (PAAs), which is associated with serious morbidity and mortality. All fatalities reported in HSS resulted from unpredictable fatal suffocating hemoptysis. Therefore, it is necessary to recognize pulmonary complications at an early stage of the disease.

**Objectives:**

The aims of this study are to develop a reference atlas of images depicting the characteristic features of HSS by computed tomography pulmonary angiography (CTPA). To make a guide for physicians by developing a classification of PAAs according to the severity and risk of complications associated with each distinct lesion type.

**Methods:**

The Members of the HSS International Study Group (HSSISG) collected 42 cases, with high-quality CTPA images in one radiology station and made reconstructions from the source images. These detailed CTPA studies were reviewed for final image selection and approved by HSSISG board members. We classified these findings according to the clinical course of the patients.

**Results:**

This atlas describes the CTPA images that best define the wide spectrum of pulmonary vasculitis observed in HSS. Pulmonary aneurysms were classified into six radiographic patterns: from true stable PAA with adherent in-situ thrombosis to unstable leaking PAA, BAA and/or PAP with loss of aneurysmal wall definition (most prone to rupture), also CTPA images demonstrating right ventricular strain and intracardiac thrombosis.

**Conclusion:**

The HSSISG reference atlas is a guide for physicians regarding the CTPA radiological findings, essential for early diagnosis and management of HSS-related pulmonary vasculitis.

**Table:** 

**Key Points** • *The Hughes-Stovin syndrome (HSS) is a systemic vasculitis characterized by extensive vascular thrombosis and pulmonary artery aneurysms (PAAs) that can lead to significant morbidity and mortality.* • *All fatalities reported in HSS were related to unpredictable massive hemoptysis; therefore, it is critical to recognize pulmonary complications at an early stage of the disease.* • *The HSS International Study Group reference atlas classifies pulmonary vasculitis in HSS at 6 different stages of the disease process and defines the different radiological patterns of pulmonary vasculitis notably pulmonary artery aneurysms, as detected by computed tomography pulmonary angiography (CTPA).* *• The main aim of the classification is to make a guide for physicians about this rare syndrome. Such a scheme has never been reached before since the first description of the syndrome by Hughes and Stovin since 1959. This classification will form the basis for future recommendations regarding diagnosis and treatment of this syndrome.*

## Introduction

The Hughes-Stovin syndrome (HSS) was named after two British physicians in 1959 John Patterson Hughes and Peter George Ingle Stovin. They described two male patients with systemic illness, recurrent deep venous thrombosis (DVT), and segmental pulmonary artery aneurysms (PAA) with recurrent hemoptysis [1]. Both patients died of sudden massive suffocative hemoptysis. At autopsy, the walls of the PAA showed heavy infiltration with inflammatory cells (lymphocytes, plasma cells, and foam cells) and segmental destruction of the elastic and muscular layers. A large “adherent thrombus” was also found in two upper lobe segmental arteries. Similar clinical associations were observed in 1912 by Beattie and Hall, who described a previously healthy man who died suddenly from profuse suffocative hemoptysis [2].Upon autopsy, an aneurysm was found to have ruptured into the adjacent lung tissue, ulcerating in the main branch of the right bronchus and bulging into the right side of the pericardium. These findings indicated “extra-luminal” extension of this inflammatory process [2].

Early case reports typically describe male HSS patients aged between 14 and 37 years, with patient survival ranging from several months to 8 years after disease onset [1–9]. All fatalities in HSS were due to suffocating massive hemoptysis. None of these patients received immunomodulators and neither the etiology nor definitive treatment was established at this time [10]. In addition to true PAA in HSS, some older reports noted pulmonary artery pseudo-aneurysms (PAPs) with destruction of a large portion of the false aneurysm’s wall and direct communication from the lumen to the adjacent bronchus, which eventually resulted in fatal hemoptysis [7–9]. These classical autopsy findings can currently be evaluated at an earlier stage using computed tomography pulmonary angiography (CTPA) imaging, which also may provide opportunities for early, targeted management of these pulmonary lesions [10–41].

The last 3 years we investigated in PubMed and other search engines who had published worldwide in the field of HSS. These authors were invited to join the HSS International Study Group (HSSISG) and almost all agreed to participate. In order to better delineate the clinical-radiological correlations observed in HSS, the Study Group recently reviewed the clinical presentation, disease course, and detailed CTPA imaging results of 57 HSS patients [10]. We observed that intra-aneurysmal thrombi in patients with HSS-related pulmonary vasculitis can evolve in situ due to the underlying arterial wall vasculitis and activation of the coagulation cascade. If untreated, this may lead to penetration of the intra-luminal thrombus through the inflamed aneurysmal wall and extension of the inflammatory process into an adjacent bronchus. The latter may result in the formation of a pulmonary artery pseudoaneurysm (PAP) with fatal hemoptysis. In one case, a patient died after PAA involving the main pulmonary artery (PA), ruptured into the pericardial space [4]. Overall, the main cause of death in HSS is typically fatal hemoptysis due to PAA or PAP rupture [1–9, 23, 29, 30, 35] as a result of unremitting pulmonary vasculitis. This clearly indicates that early detection and close subsequent surveillance of pulmonary vascular lesions should be a top priority in the management of HSS patients [10].

The objective of the current report is to create a comprehensive and illustrative reference atlas that demonstrates all possible CTPA pulmonary imaging findings that may be observed in HSS-related pulmonary vasculitis at various disease stages. This reference atlas will assist treating physicians to reach the diagnosis and precisely characterize the specific pulmonary vascular lesions at an early stage of the disease process. In addition, the atlas defines the most serious CTPA radiological signs that warrant special consideration and show the clinicians when urgent interventions are necessary in order to avoid adverse or fatal outcomes.

## Patients and methods

The reference atlas was created after critical and extensive review of detailed CTPA studies from 31 previously reported HSS case reports, representing a total of 42 HSS patients (11–41). Due to a lack of diagnostic criteria for HSS, in previous reports, diagnosis of HSS was based on the presence of typical disease features such as (a) widespread recurrent vasculo-occlusive disease in the form of recurrent superficial thrombophlebitis, DVT, cerebral venous sinus thrombosis (CVST), intracardiac thrombosis, or arterial thrombosis; (b) pulmonary vasculitis with typical CTPA features, notably pulmonary artery aneurysms and in situ thrombosis and/or bronchial artery aneurysms; and (c) normal coagulation profiles.

**Fig. 1:**
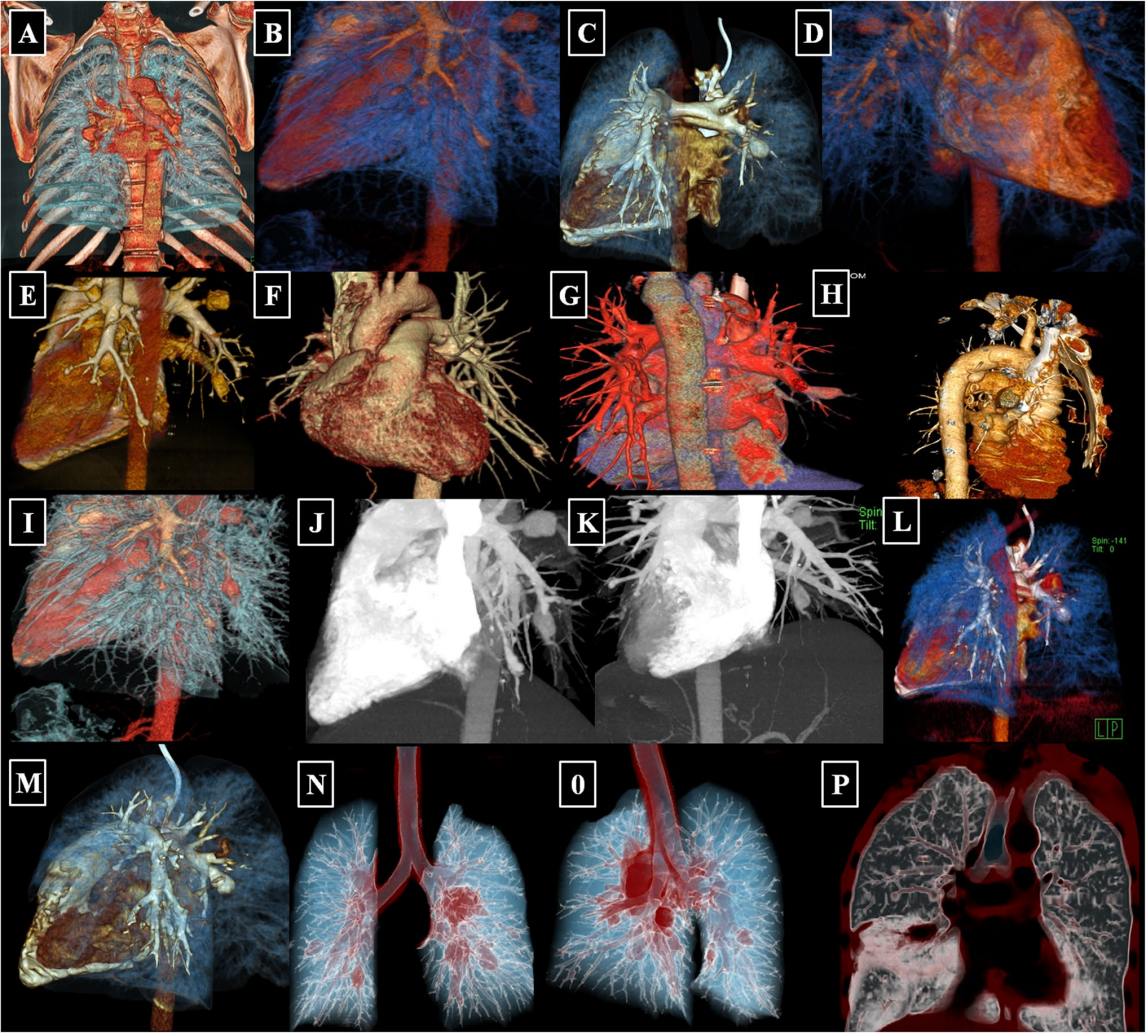
Multiple 3D MIP reconstructed CTPA images from different patients. **A** Right central PAA. **B**–**G** Examples of central PAA in different patients. **H** Bronchial artery aneurysm (BAA). **I**–**M** Different cases of central and peripheral PAA. **N**–**O** Bilateral central PAA of the same patient. **P** Leaking right basal true PAA in a different case

All authors of the HSS working group agreed to cooperate with the study and made their CT studies available that were uploaded to a Siemens (Syngo.via) CT workstation. The uploaded source images were interpreted in different windows such as lung and mediastinal windows in both pre- and post-contrast phases and further analyzed. Software was applied to make new reconstructions from the source images, e.g., multiplanar reconstruction *(*MPR), maximum intensity projection (MIP), and three dimensional volume rendering technique (3D-VRT) (Fig. 1).

These images were critically reviewed and consensus was reached, in order to determine the most relevant CTPA images to be included. Image selection was based on image quality and content, ensuring that the most relevant CTPA radiological signs were included that could delineate all possible pulmonary lesions in HSS at various stages of development. After deliberation and image analysis, final decisions were reached and approved by all HSSISG board members. In the case of disagreement on any of the findings, further rounds of reviews were completed until final consensus was reached by all HSSISG members.

### Interpretation of computed tomography pulmonary angiography findings

The available CTPA images were reviewed and interpreted with special emphasis on the following items:

I Adherent intra-aneurysmal in situ thrombosis with or without aneurysmal wall enhancement (on post-contrast CTPA images) proximal or distal to site of the adherent thrombus
II True “stable” PAA at different stages of development and diameter that involve various PA branches including the main, lobar, and segmental PA, as well as bronchial artery aneurysms (BAA).
III Leaking “unstable” PAA or leaking BAA with loss of aneurysmal wall definition, signaling impending rupture.
IV Stable pulmonary artery pseudo-aneurysms (PAP).
V Unstable PAP.
VI Intracardiac thrombosis and right ventricular strain (interventricular septum flattening secondary to the altered pulmonary hemodynamics).

In addition, 3D-VRT and MIP reconstructions were performed in some patients (Figure 1).

### Statistical analysis

Data analysis was performed using SPSS version 15.0.1. Continuous data was described using mean (SD) and categorical data using frequencies and percentages. Regression analysis was performed to examine the relationships between fatal outcomes and other relevant independent variables. A *P*-value < 0.05 was considered statistically significant.

## Results

Demographic features, clinical presentations, vascular thrombotic events, laboratory findings, CTPA findings, and lines of treatment among the studied group of patients are presented in Table 1. Regression analysis showed a significant positive association between fatal outcomes as a dependent variable and ruptured PAAs (*P* = 0.002) as an independent variable. Significant negative associations were observed between fatal outcome versus PACE (*P* < 0.004) and versus treatment with combined immunomodulators (*P* < 0.001). While no significant associations were observed between fatal outcomes versus age (*P* = 0.188), gender (*P* = 0.113), and anti-coagulation therapy (*P* = 0.076).

**Table 1:** Demographic, clinical presentations, arterial and/or venous thrombotic complications, lines of treatment, and fatal outcomes among the studied group of HSS patients

HSS patients (*n* = 42)	
Variables	Values
Male/female	30(71.4)/12(28.6)
Age (years) Median (IQR)	34.83 ± 12.396 35(17.50)
Age at onset (years) Median (IQR)	31.83 ± 10.467 31.5(16.25)
Disease duration (months) Median (IQR)	56.50 ± 65.431 31.5(54)
Fever	27(64.3)
Weight loss	14(33.3)
Cough	39(92.9)
Dyspnea	34(81)
Pleuritic chest pain	5(11.9)
Hemoptysis (mL/24 h)	37(88.1)
None	5(11.9)
Mild hemoptysis (< 20 mL/24 h)	14(33.3)
Moderate hemoptysis (20 to 600 mL/24 h)	12(28.6)
Massive hemoptysis (> 600 mL/24 h)	11(26.2)
DVT	34(81)
IVC thrombosis	12(28.6)
Recurrent superficial thrombophlebitis	25(59.5)
Cerebral venous sinus thrombosis	3(7.1)
Intracardiac thrombosis	8(19.0)
Arterial thrombosis	6(14.3)
ESR 1st hour (mm/h)	50.167 ± 25.18
CRP (mg/dl)	15.205 ± 15.33
Hemoglobin	11.23 ± 1.68
WBCs	8.6738 ± 4.103
Platelets count	319.74 ± 92.334
Oral steroids therapy	39(92.9)
Oral azathioprine therapy	18(42.9)
Monthly intravenous pulse cyclophosphamide therapy	19(45.2)
Anti-*TNF* inhibitors	3(7.1)
Combined immunosuppressants (oral steroids combined with either azathioprine or cyclophosphamide)	35(83.3)
Anticoagulation therapy	31(73.8)
Surgical lobectomy or segmentectomy	7(16.7)
PACE	8(19.0)
Lobar pulmonary artery aneurysms	35(83.3)
Segmental pulmonary artery aneurysms	26(61.9)
Bilateral pulmonary artery aneurysms	37(88.1)
Size of the largest pulmonary artery aneurysm (mm)	25.519 ± 12.318
Bronchial arterial aneurysms	4(9.5)
Ruptured pulmonary aneurysm(s)	12(28.6)
Fatal suffocative hemoptysis	5(11.9)

Data are mean ± (SD), and others are number (%); *IQR* inter quartile range, *HSS* Hughes-Stovin syndrome, *DVT* deep vein thrombosis, *IVC* inferior vena cava, *PACE* pulmonary artery coil embolization, *TNF* tumor necrosis factor

**Fig. 2:**
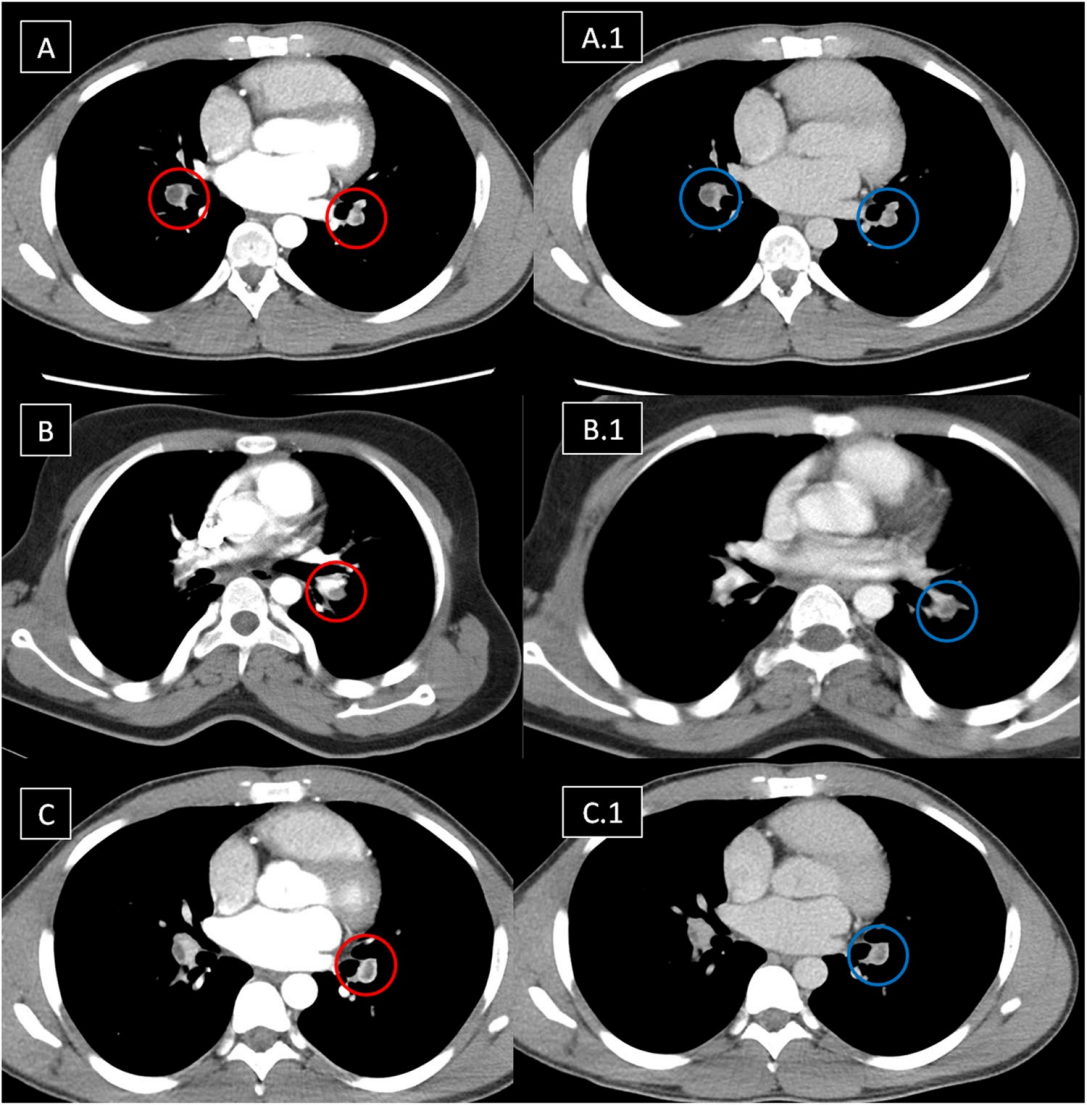
Sequential images in the arterial phase (in the left side column and labeled with red circles) and the corresponding venous phase images (in the right side column-labeled with blue circles) for 3 different patients, showing lobar PAA, in situ thrombosis with arterial mural enhancement in venous images denoting vasculitis. Variable degrees of luminal dilation represent different stages of development. (A and A1) Bilateral central pulmonary in situ thrombosis as filling defects (*red circles*) and mural enhancement in the venous phase (*blue circles*). (B and B1) Left-sided true stable PAA with large eccentric filling defect represents in situ thrombosis (*red circle*) and mural enhancement in the venous phase (*blue circle*). (C and C1): Bilateral central pulmonary in situ thrombosis as filling defects (*red circles*) and mural enhancement in the venous phase of the left side pulmonary artery with wall enhancement (*blue circle*)

**Fig. 3:**
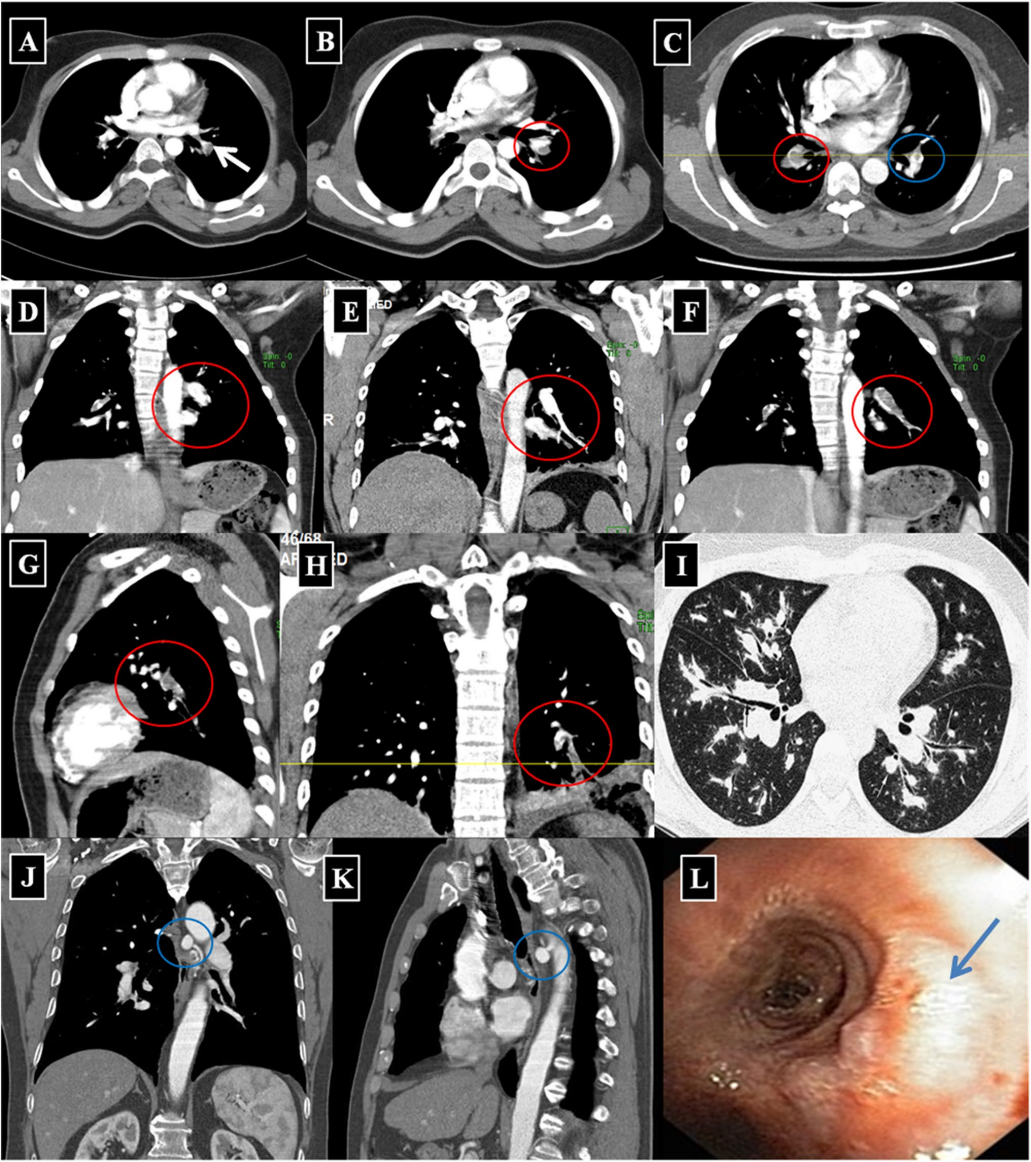
Different cases with variable bilateral lobar and segmental true stable PAA and true BAA. **A**, **B** Two different cases of left-sided true central PAA and in situ thrombosis (*white arrow in image ****A**** and red circle in image ****B***). **C** A different case of bilateral stable proximal PAA with in situ thrombosis (*red circle around the right-sided PAA and blue around the left-sided PAA*). **D**, **E** BAA in coronal reformatted images (*red circle around the BAA*). **F**–**H** Coronal and sagittal reformatted images representing left lower lobe stable PAA with in situ thrombosis. **I** Mild perivascular pulmonary parenchymal changes in bilateral stable PAA. **J**, **K** Stable BAA (*blue circle around the left-sided BAA*). **L** Bronchoscopy for the same patient revealed a pulsatile prominence in the right main bronchus (*blue arrow*)

**Fig. 4:**
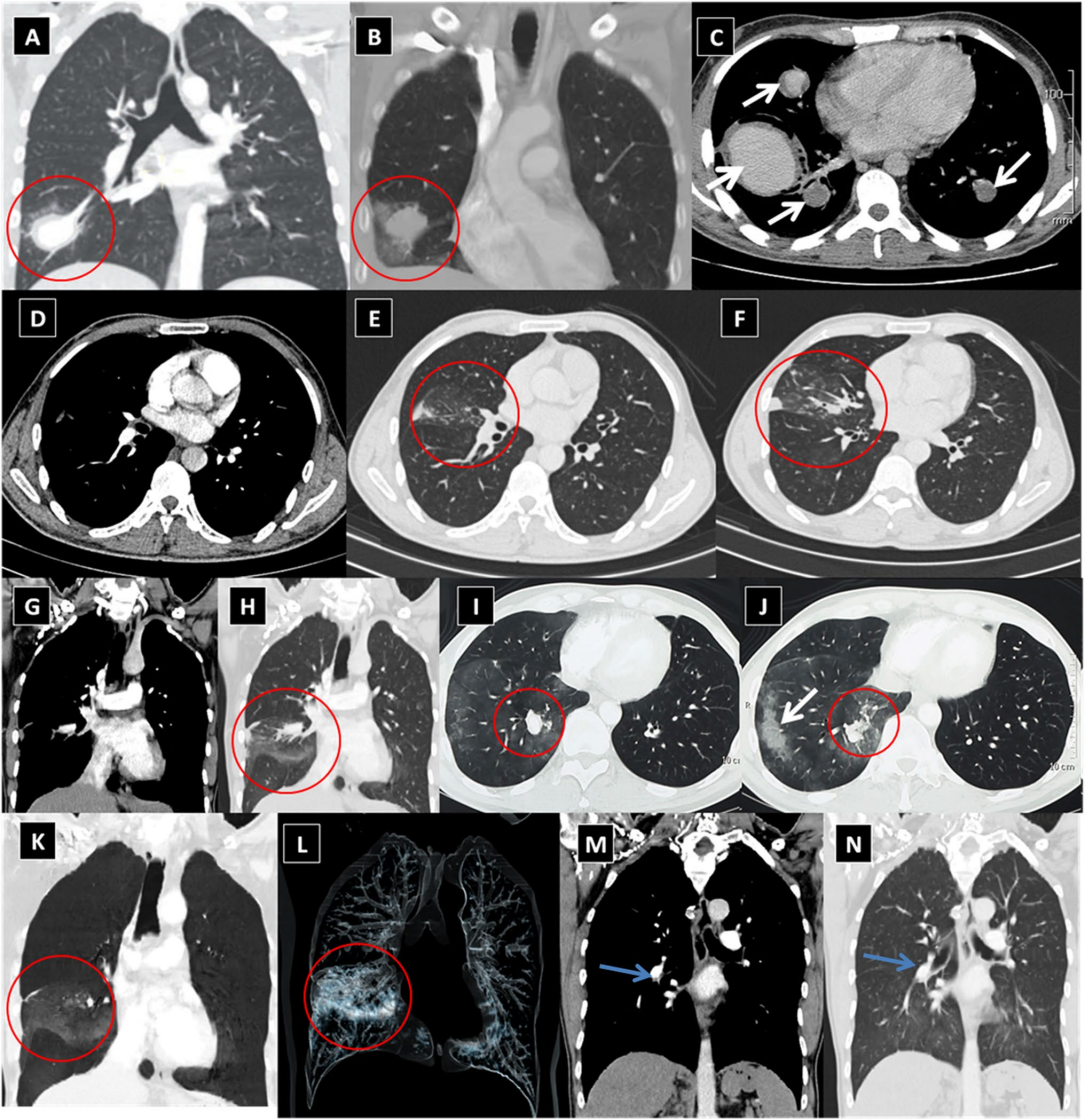
Examples of variable-sized lobar and segmental unstable leaking PAAs with adjacent parenchymal lung opacification and veiling infiltrates due to acute aneurysmal leakage. These include axial and coronal reformatted images of the lung and mediastinal windows for true leaking PAAs. **A**, **B** Two different patients with right lower lobe unstable PAA (*red circles*) with subtle leakage and ground glass opacification of the right middle lung lobe. **C** Another patient with bilateral central unstable PAA (*white arrows*). **D**–**J** Different examples of right proximal and peripheral true PAA with peripheral leakage and ground-glass opacification (*red circles*). **K**–**N** Coronal reformatted images of right central and peripheral leaking true PAA in volumetric MIP projections (**K**, **L**, red circle), mediastinal (**M**, blue arrow), and lung windows (**N**, blue arrow)

**Fig. 5:**
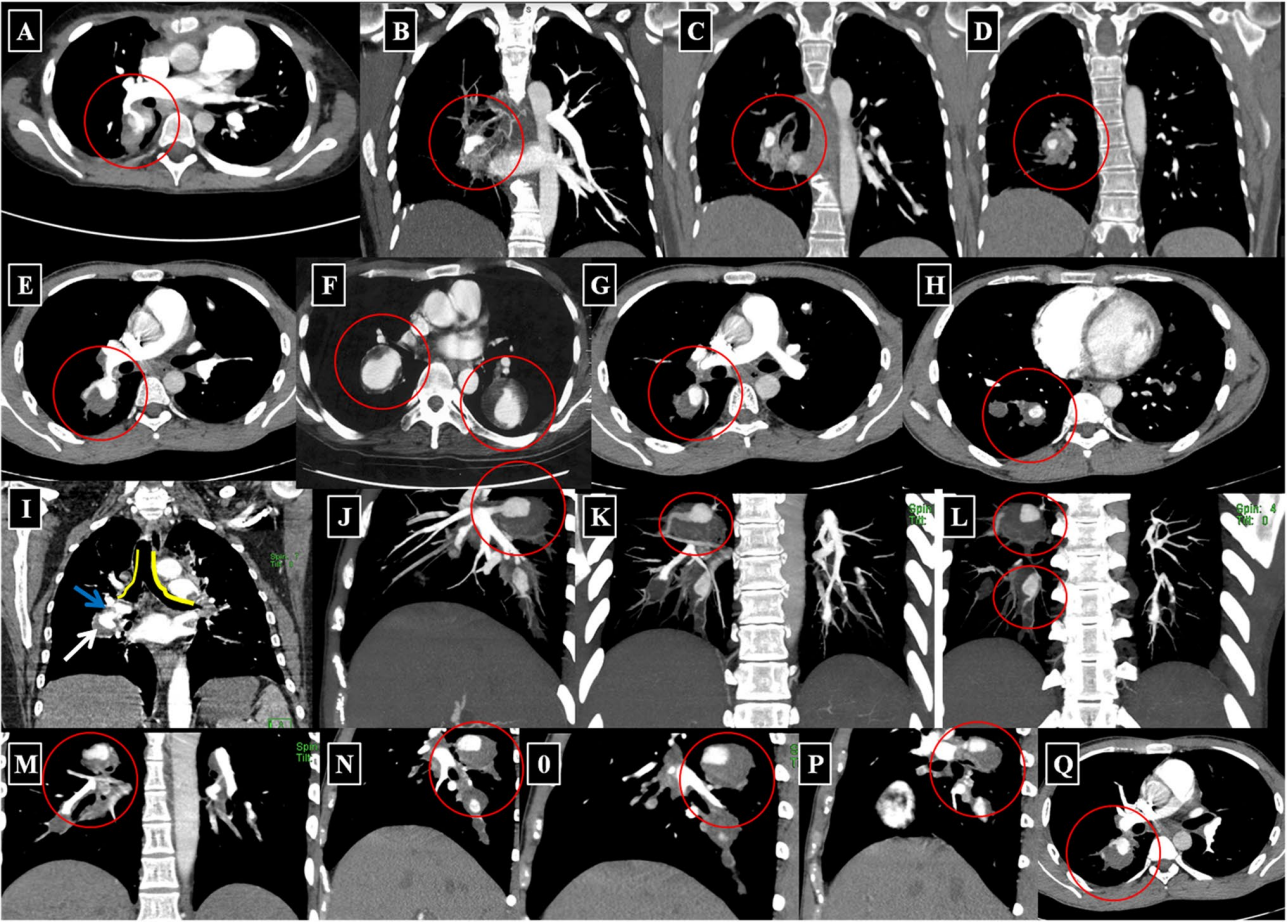
Examples of variable-sized pulmonary artery pseudoaneurysms (PAP) lesions involving the central and segmental pulmonary arterial branches. Axial, sagittal, and coronal CTPA reformatted images of mediastinal windows, which allowed for sharp demarcation of PAP with eccentric thrombosis. **A**–**E** Right-sided proximal PAP with eccentric thrombosis. **F** A different case of bibasal PAP. **G**–**H** Two cases of right-sided basal proximal PAP with ectatic lumens. **I** Contrast-filled lumen (*blue arrow*), surrounded by a hypodense area of eccentric thrombus (*white arrow*), noting the relationship between the PAP lesion and the adjacent bronchus. **J**–**Q** Different cases of right-sided basal proximal and peripheral PAP with ectatic lumens and eccentric mural thrombosis. (*Lesions are labeled with red circles for the purpose of illustration*)

**Fig. 6:**
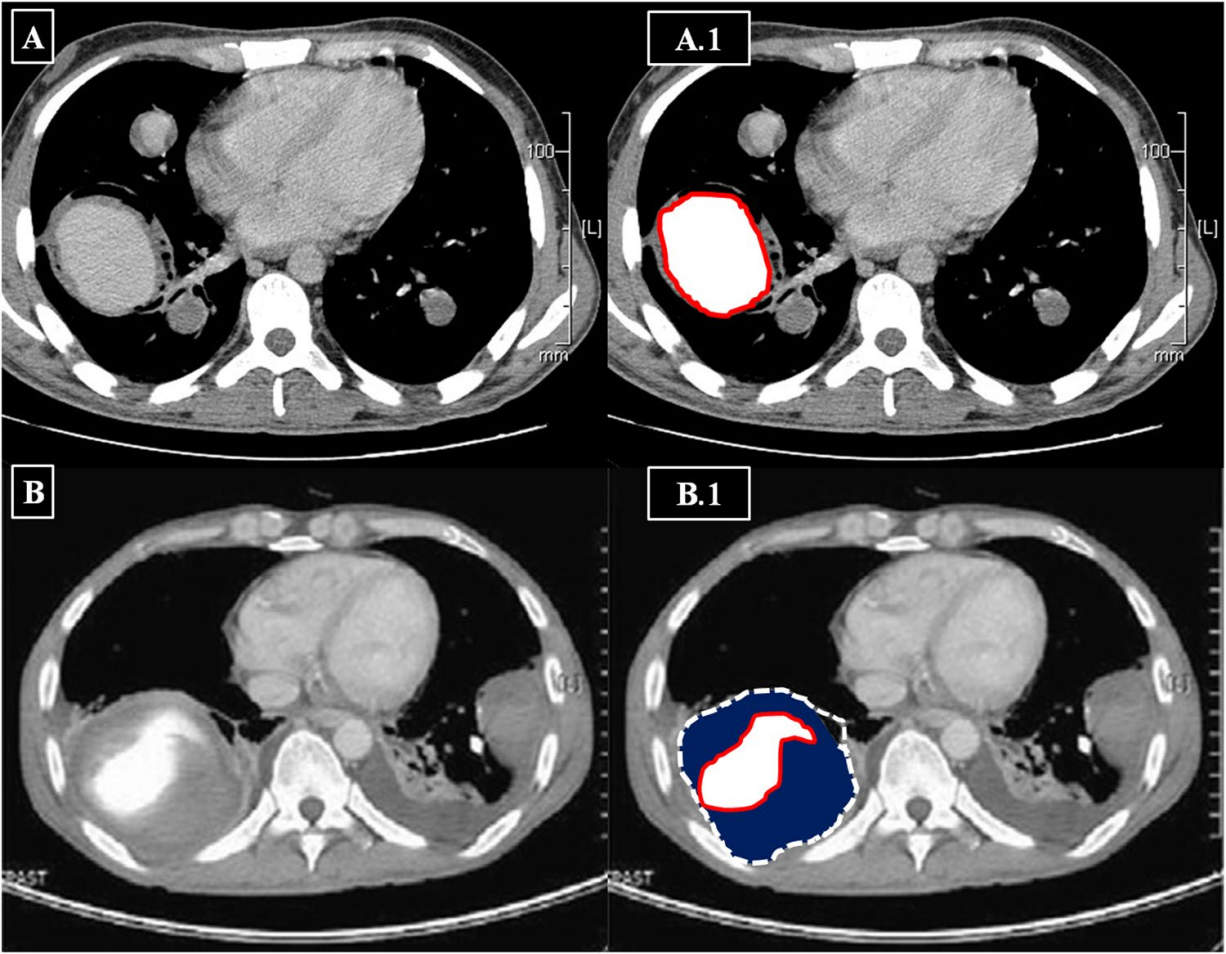
Diagrammatic representation of a large true PAA in two different patients. (A and A-1) Axial CTPA shows a bibasal PAA with in-situ thrombosis and a massive large right-sided basal peripheral PAA with an intact aneurysmal wall and well demarcation (*Red line representing the mural wall of the true PAA in A.1*). (B and B-1) Active leakage of the right-sided basal large PAP with concentric mural thrombus, bibasal consolidation, and intra-pleural hemorrhagic collection (*red line encircling the true contrast-filled lumen of PAP in B*.*1*, *plus an interrupted white line representing the false wall of the aneurysm*)

**Fig. 7:**
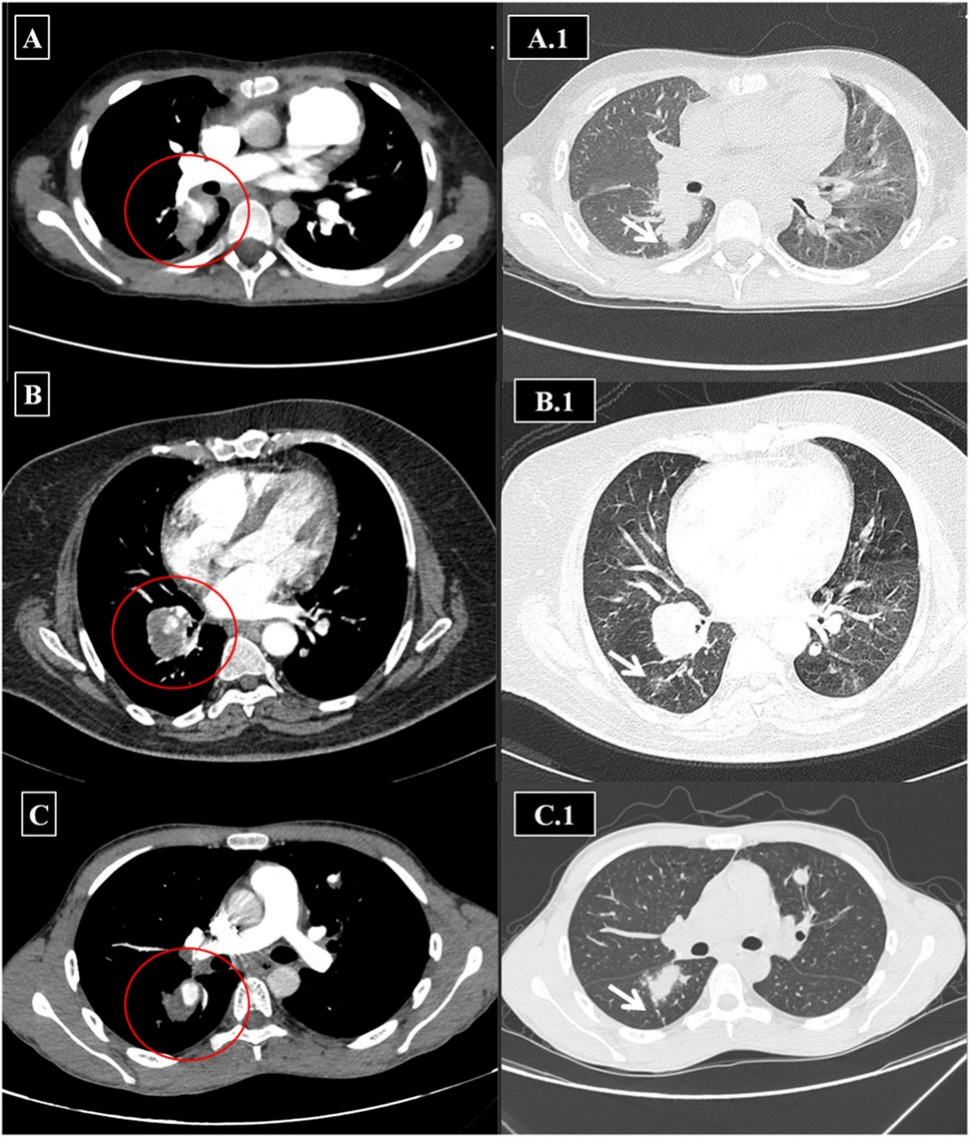
Examples of disturbed PAP in different patients. Selected axial images of CTPA in the mediastinal and same level lung window for three different patients. (A) Unstable PAP of the right main pulmonary artery posterior wall with eccentric posterior hypodense thrombus entangling the contrast filled aneurysm. (A.1) Lung window at the same level as the previous image with evidence of subtle perianeurysmal airspace consolidation and posterior pleuro-pulmonary reactive thickening due to the unstable PAP. (B) Right lower lobe unstable PAP with a posterior circumferential thrombus surrounding the contrast-filled eccentric lumen and subtle developing right-sided posterior ground-glass opacification seen at the corresponding (B.1) lung window. (C) Right lower lobe unstable PAP with eccentric thrombus surrounding the sharply demarcated contrast-filled eccentric lumen. The corresponding (C.1) lung window image also shows patchy lung consolidation in the perianeurysmal parenchyma (note that there is a left lingular stable PAA with an eccentric in-situ thrombus seen in the same patient). (Lesions are labeled in the pulmonary vascular window with red circles and in the lung window with white arrows for the purpose of illustration)

After extensive review of available CTPA images, a final consensus was reached by HSSISG members regarding the radiological definitions of each individual lesion. We present the following radiological definitions and classifications relative to CTPA imaging findings:

I Aneurysmal wall enhancement on post-contrast CTPA
  Arterial wall enhancement is the earliest radiological sign in HSS-related pulmonary vasculitis. It is frequently observed during early stages of the disease process. Arterial wall enhancement is radiologically defined as an “enhancing aneurysmal wall,” which is typically visualized in the mediastinal window in sequential arterial and venous post-contrast phases (Fig. 2).
II True “stable” PAA
  PAA is generally defined as a dilatation involving all three layers of the vessel wall and can affect all PA branches, including the main PA and its branches, e.g., lobar and segmental. True stable PAA is defined radiologically as an “aneurysmal lesion (contrast filled) of the affected PA branch with a well-defined aneurysmal wall and associated with intra-aneurysmal adherent in-situ thrombosis (filling defects) without any perianeurysmal parenchymal ground-glass opacification (GGO), which would be suggestive of an extra-luminal acute leak (best visualized in the lung window)” (Fig. 3).
III Unstable leaking true PAA (“acute phase”)
  Radiologically unstable PAA is defined as “aneurysm formation (contrast-filled) of the affected PA branch with loss of aneurysmal wall definition and perianeurysmal alveolar hemorrhage (ground-glass opacification and/or consolidation) with ‘air-bronchograms’.” The latter refers to air-filled bronchi (dark) being made visible by the opacification of surrounding alveoli (gray/white). This finding is almost always caused by a pathologic airspace/alveolar process in the adjacent surrounding lung parenchyma, which can occur as a result of hemorrhage (best visualized in the lung window) (Fig. 4).
IV Pulmonary artery pseudoaneurysm (PAP) (“chronic phase”)
  A PAP is defined radiologically as “sharply demarcated contrast-filled aneurysmal lesions with a variably sized marginal hypodense perianeurysmal component (‘marginal thrombosis’) entangling a contrast-filled ectatic lumen.” The lesion is not associated with adjacent ground glass opacification or frank consolidation, distinguishing it from leaking PAA or PAP. Air bronchograms (air-filled bronchi/bronchiole) can be associated within the hypodense component (“marginal thrombosis”) (Fig. 5). The radiological and morphological differences between true PAA versus PAP are illustrated in (Fig. 6).
V Unstable PAP
  Unstable PAP is defined radiologically as “sharply demarcated contrast-filled aneurysmal lesions with a variably sized marginal hypodense perianeurysmal component (i.e. ‘marginal thrombosis’) entangling the sharply demarcated contrast-filled ectatic lumen plus adjacent ground glass opacification or frank consolidation due to active hemorrhage from the leaking ectatic lumen.” The air bronchogram (air-filled bronchi/bronchiole) can be associated within the hypodense component (Fig. 7).
VI Right ventricular strain (RVS) with or without intracardiac thrombosis.

**Fig. 8:**
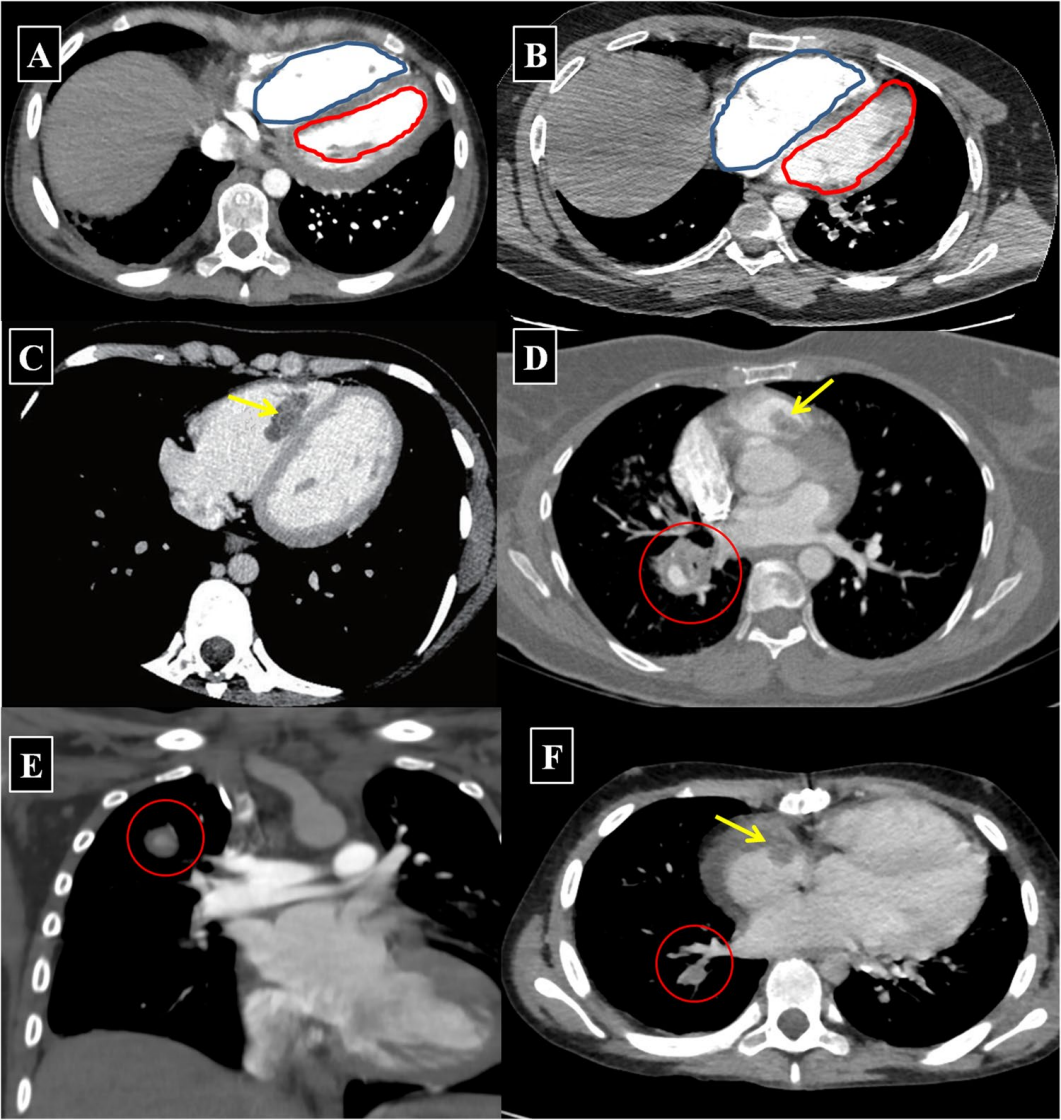
**A**, **B** Right ventricular strain is demonstrated as an increased transverse cross-sectional diameter of the right ventricle (outlined by a blue line) compared to the left ventricle (outlined by a red line). Selected axial CTPA images of true right-sided PAA (*red circles in image ****D****–****F***) with intra-cardiac thrombus within the right ventricle (*yellow arrows*, *image ****C**** and ****D***) and within the right atrium (*yellow arrow in image ****F***)

RVS is defined radiologically by CTPA as “interventricular septal flattening or paradoxical interventricular septal bowing towards the left ventricle, which occurs secondary to the altered pulmonary hemodynamics in the context of pulmonary hypertension.” In addition, RVS is characterized by a right ventricle size that exceeds that of the left ventricle. Intracardiac thrombosis is defined by CTPA as a low attenuation non-enhancing filling defect in the involved cardiac chamber(s) (Fig. 8).

## Discussion

The atlas gives a state of the art of the CT pulmonary angiographic findings in HSS patients. Given the lack of a diagnostic criteria for HSS, the diagnosis is made based on the typical clinical features of the disease including widespread vascular occlusive disease (venous and/or arterial), recurrent superficial thrombophlebitis, and a normal coagulation profile. An integral component of the diagnosis of HSS is the characteristic CTPA findings of pulmonary vasculitis, which include PAA, adherent intra-aneurysmal in situ thrombosis, and aneurysmal wall enhancement on post-contrast CTPA, BAA, and PAP. These radiological features were recently highlighted by our group in the first report from the HSSISG [10].

Despite the classic clinical and characteristic CTPA findings of HSS, delayed diagnosis is common and misdiagnosis for isolated PTE may occur [10–41]. To facilitate earlier diagnosis and reduce the potential morbidity associated with misdiagnosis, the HSSISG assembled CTPA images into this reference atlas that we believe demonstrate all potential pulmonary complications associated with HSS. Our rationale was to provide the most illustrative and reliable collection of CTPA images, defining pulmonary vasculitis in HSS at different stages of the disease process, and to classify the different radiological patterns of pulmonary vasculitis and aneurysm formation. These classifications were developed according to lesion severity and the potential morbidity associated with each individual lesion. PAAs are considered to be one of the most consistent signs of pulmonary vasculitis by CTPA imaging in both HSS [10–41] and Behçet’s disease (BD) [42]. In our opinion, the earliest CTPA sign of HSS-related pulmonary vasculitis is arterial wall enhancement of the mural wall of the PAA (Fig. 2).This finding is typically observed early at disease onset and in new developing lesions. This specifically reflects arterial mural wall inflammation due to the underlying vasculitic process [10]. Pirani et al. documented this histopathological pattern in 1949 [5], finding that the wall of a ruptured PAA showed significant lymphocytic infiltrations and disruption of the muscular layer. Similar results were reported by Kopp and Green in 1962 [6], Kirk and Seal in1964 [7], and Kinjo et al. in 1978 [9]. More recently, Robinson et al. (2018) described a histopathological pattern of necrotizing lymphocytic vasculitis with adjacent pulmonary infarction, from a resected unstable true PAA (by CTPA imaging) [28] (Fig. 4A). Persistent arterial wall inflammation of PA branches results in progressive vessel injury, and over time with exposure to the shear forces of the pulmonary circulation pressure, this can result in an outpouching of the wall, which is radiologically identified in our report as “true stable PAA” (Fig. 3). “True PAAs” are histologically defined as focal dilatation of PA branch involving all three layers of the arterial wall including tunica intima, tunica media, and tunica adventitia [43].

Since HSS is a systemic vasculitis, the same inflammatory process may also affect the bronchial arteries. Hughes and Stovin [1] found that the bronchial arteries were significantly affected in their cases, with complete loss of the elastic lamellae, thinning of the media, and distortion of the lumen by conspicuous cushions of edematous sparsely cellular tissue in their autopsy study. Furthermore, BAAs in HSS have also been documented in the recent literature with the use of CTPA [15, 29].

The pulmonary and bronchial arteries are two distinct vascular networks that supply the lungs. The pulmonary arteries supply 99% of the blood flow to the lungs and engage in gas exchange at the alveolar capillary membrane, carrying deoxygenated blood at low pressure [44]. The bronchial arteries, on the other hand, provide oxygenated blood to the lungs at a sixfold higher pressure than the pulmonary arteries, and they are linked to the pulmonary arteries by multiple microvascular anastomoses [45]. Due to their smaller diameter, the bronchial arteries can develop high resistance, low capacitance, and less distensible circulation. In the context of reduced pulmonary blood flow, the bronchial circulation and other collateral vessels can hypertrophy in order to sustain blood flow to areas of ischemic lung and engage in gas exchange through systemic-pulmonary arterial anastomoses [46, 47]. The total systemic cardiac output to the bronchial arteries may increase from 1 to 18–30% in certain disease states, such as pulmonary thromboembolism (PTE) or widespread in situ thrombosis in HSS-related pulmonary vasculitis [44]. Increased bronchial artery pressure can contribute to BAA formation. BAA can subsequently result in compression, deformation, or even destruction of bronchial wall, which can be occasionally seen during bronchoscopy (Fig. 3L).

PAA in HSS can evolve over time to acquire different morphological patterns. These patterns are observable by CTPA, which can be used to define the size and stability of PAA. Leakage from PAA is a critical event during the disease course and can result in either of two distinct scenarios. The first scenario is an acute leaking process which turns a “stable true PAA” into a “leaking unstable PAA” with loss of aneurysmal wall definition and adjacent parenchymal hemorrhage (Fig. 4). The latter may be aggravated if the patient is already receiving anti-coagulation therapy for peripheral vascular occlusive disease, which is known to complicate many cases of HSS [10]. The second scenario is more serious and unpredictable and tends to run a slower chronic course. In this scenario, blood slowly and repeatedly leaks through the inflamed aneurysmal wall, to surround the exterior wall of the PAA. Over time, this slowly accumulates and acquires a false wall (i.e., formation of a PAP lesion) that can indent or invade adjacent bronchi. This morphological progression is illustrated by CTPA as hypodense non-enhancing marginal thrombus surrounding the exterior wall of the ectatic well-defined contrast-filled lumen (Fig. 5). In both scenarios, extra-luminal extension of the inflammatory process may lead to rupture into an adjacent bronchus and fatal hemoptysis. This was described by Hughes and Stovin themselves as being unpredictable massive suffocative fatal hemoptysis [1].

From the therapeutic point of view, this classification is very important. Patients with asymptomatic stable PAAs that are multiple, confluent, and/or involve both lung fields may benefit from combined immunomodulators to induce and maintain disease remission. For this subgroup of patients, embolization or surgical resection procedures may not be technically feasible or warranted, given the responsiveness of the lesions to immunosuppressive treatment. On the other hand, indications for procedural management (typically by pulmonary artery coil embolization, or open thoracotomy for certain high-risk lesions) include large PAA size (i.e., > 30-mm peripheral PAA), rapidly increasing PAA size, and leaking unstable PAA or PAP. The presence of pulmonary artery hypertension is also an important risk factor for subsequent aneurysm rupture/dissection, and should be considered a relative indication for procedural management to stabilize the lesion. Unstable leaking true PAA or PAP requires urgent procedural stabilization, even in the context of only mild symptoms. This concept was supported by the histopathological results reported in 1964 by Kirk and Seal [7], who reported a false aneurysm (PAP) with a segmental disruption of the elastica of the PA at its origin. The clot itself was largely extra-luminal and much of its wall was formed by an expanded “false wall” of the adjacent bronchus. The organizing thrombus was separated from the bronchial lumen by a thin layer of respiratory epithelium, and squamous metaplasia had occurred in places. The edge of the false aneurysm was encroaching on a bronchial lumen and a branch of the PA showed disruption of the tunica elastica. This highlights the importance of securing a potentially unstable PAP with PACE, and the need to discontinue anticoagulation and subsequently control the disease with combined immunomodulators, to prevent further progression of the pulmonary vasculitis and new aneurysm formation.

In our recent report, we found that early initiation of combined immunomodulators was associated with a significantly lower risk of death (*P* < 0.001). Individuals that underwent PAA embolization and/or coiling also had a lower risk of death, but this was not statistically significant (*P* = 0.06).These interventions are indicated on an urgent or emergent basis in cases of unstable leaking PAA or PAP, which would be at excessive risk of rupture during the interim period before immunosuppression can induce remission of the pulmonary vasculitis [10].

Finally, CTPA has the potential to detect RVS related to alerted pulmonary hemodynamics in cases of widespread in situ thrombosis of the PA branches, as well as the potential to detect intracardiac thrombosis in HSS. If RVS is detected during the initial evaluation of a patient with HSS, full cardiac assessment is recommended. The concept of RVS secondary to altered pulmonary hemodynamics is well-established in PTE. CTPA is recommended as a more accurate tool for assessment of the severity of obstruction of the pulmonary vascular bed and its secondary consequences, including RVS and upstream consequences in abdominal and thoracic venous pathways [48].

Based on currently available data, PTE- and HSS-related in situ thrombosis appear to be pathophysiologically distinct entities. PTE is caused by embolization of a thrombus from the venous periphery to the lungs. In HSS, the pulmonary arterial thrombus typically evolves in situ due to the underlying pulmonary arterial wall vasculitic process. However, pulmonary vascular bed obstruction and altered pulmonary hemodynamics will ultimately lead to RVS in both clinical entities. Intra-cardiac thrombosis also seems different from the pathophysiological point of view in both conditions. PTE happens when blood clots (type A thrombi) which are serpiginous and highly mobile travel through the blood stream from the deep venous system. These can be captured within the right side of the heart (thrombus in-transit) before reaching the lung. In HSS, the underlying cause of intra-cardiac thrombosis is still not clearly understood despite the reporting of multiple cases [22, 32, 38]. In one patient with HSS and widespread in situ thrombosis, an echocardiogram showed tricuspid annular plane systolic excursion (TAPSE 14 mm) and moderate tricuspid regurgitation, with an estimated systolic pulmonary artery pressure of 45 mmHg, all indicative of possible RV dysfunction [15]. This potential association needs to be further investigated in future studies of HSS-associated intracardiac thrombosis.

We include detailed descriptions of the CTPA imaging findings of all major pulmonary complications in HSS. By using CTPA imaging techniques, we objectively classified PAA into stable PAA, unstable leaking PAA, and PAP. This classification is important when considering optimal treatment modalities. PAA embolization/coiling is recommended for patients who have or are at risk of major bleeding in the 2018 update of the European League Against Rheumatism (EULAR) recommendations for BD management [49]. In the current study, the risk of major bleeding was qualified using a novel radiological classification to describe various morphological and radiological patterns of PAA in HSS. The objective of this classification is to facilitate risk assessment regarding the threat posed by individual pulmonary lesions in HSS for subsequent rupture and/or fatal hemoptysis. Improved understanding of the prognostic implications of different imaging findings may help determine the need for and urgency of procedural interventions.

A limitation of the current study is that we included a relatively small number of patients (*n* = 42). In this regard, the results and conclusions should be interpreted with caution.

A strength of our study is that we could include most of the published cases worldwide that applied CTPA imaging; it would hardly be possible to find a larger series.

So the atlas is important for rheumatologists, internal medicine specialists, and radiologists to be able to classify the CTPA findings in HSS patients and to give appropriate treatment and prevent unnecessary deaths.

## Conclusions

1. The main aim of the classification is to make a guide for physicians about this rare syndrome. Such a scheme has never been reached before since the first description of the syndrome by Hughes and Stovin since 1959.
2. The HSSISG reference atlas is a state of the art comprehensive and illustrative manual guide that can assist physicians to identify and classify patients with HSS at an early stage of disease. The reference atlas highlights the most serious CTPA signs that warrant special consideration, given the associated risk of future rupture and fatal hemoptysis.
3. The atlas covers the following components: aneurysmal wall enhancement on post contrast CTPA images as an early sign of pulmonary vasculitis and adherent intra-aneurysmal in situ thrombosis. The atlas classifies HSS-related pulmonary vasculitis with aneurysm formation into the following categories: true PAA at different stages of development, BAA, and PAP. Leaking unstable PAA and/or BAA and unstable PAP lesions pose the greatest impending risk of rupture.
4. We consider that radiological evaluation of the proximity and structural associations between PAA and PAP with adjacent bronchi is critical to the process of assessing the risk of subsequent rupture and fatal outcomes in HSS-related vasculitis. This evaluation should guide decision-making regarding the need for interventional procedures to secure individual pulmonary lesions.

## Abbreviations

HSS: Hughes-Stovin syndrome
CTPA: Computed tomography pulmonary angiography
DVT: Deep venous thrombosis
PAAs: Pulmonary artery aneurysms
BAA: Bronchial artery aneurysms
PAPs: Pulmonary artery pseudo-aneurysms
MPR: Multiplanar reconstruction
3D-VRT: Three-dimensional volume rendering technique
GGO: Ground-glass opacification
RVS: Right ventricular strain
PTE: Pulmonary thromboembolism
BD: Behçet’s disease
PA: Pulmonary artery
PACE: Pulmonary artery coil embolization
EULAR: European League Against Rheumatism
